# Mammalian Models of Traumatic Brain Injury and a Place for *Drosophila* in TBI Research

**DOI:** 10.3389/fnins.2019.00409

**Published:** 2019-04-26

**Authors:** Ekta J. Shah, Katherine Gurdziel, Douglas M. Ruden

**Affiliations:** ^1^Department of Pharmacology, Wayne State University, Detroit, MI, United States; ^2^Department of Obstetrics and Gynecology, Wayne State University, Detroit, MI, United States; ^3^Institute of Environmental Health Sciences, Wayne State University, Detroit, MI, United States

**Keywords:** *Drosophila*, traumatic brain injury, RNA-seq, stress, neurogenetics, behavioral genetics

## Abstract

Traumatic brain injury (TBI), caused by a sudden blow or jolt to the brain that disrupts normal function, is an emerging health epidemic with ∼2.5 million cases occurring annually in the United States that are severe enough to cause hospitalization or death. Most common causes of TBI include contact sports, vehicle crashes and domestic violence or war injuries. Injury to the central nervous system is one of the most consistent candidates for initiating the molecular and cellular cascades that result in Alzheimer’s disease (AD), Parkinson’s disease (PD) and amyotrophic lateral sclerosis (ALS). Not every TBI event is alike with effects varying from person to person. The majority of people recover from mild TBI within a short period of time, but repeated incidents can have deleterious long-lasting effects which depend on factors such as the number of TBIs sustained, time till medical attention, age, gender and genetics of the individual. Despite extensive research, many questions still remain regarding diagnosis, treatment, and prevention of long-term effects from TBI as well as recovery of brain function. In this review, we present an overview of TBI pathology, discuss mammalian models for TBI and focus on current methods using *Drosophila melanogaster* as a model for TBI study. The relatively small brain size (∼100,000 neurons and glia), conserved neurotransmitter signaling mechanisms and sophisticated genetics of *Drosophila* allows for cell biological, molecular and genetic analyses that are impractical in mammalian models of TBI.

## Introduction

TBI represents a major health problem affecting about 10 million people worldwide each year (Hyder et al., 2007). Although the precise number of people living with TBI-related disability is unknown, it has been estimated that about 5.3 million people live with long-term disability after being hospitalized for TBI in the US alone (Faul et al., 2010). Depending on the intensity and location of the impact, the injury can be classified as mild (mTBI), moderate or severe ranging from brief loss of consciousness to unconsciousness lasting more than 6 hours (h) (Mayfield Certified Health Info, 2018). Repetitive mTBI (rmTBI) events can have long-term consequences that adversely affect the ability of a person to perform daily activities (McAllister et al., 2001; Stalnacke et al., 2007; NINDS, 2015). Studies of outcome following mTBI and rmTBI in adults have shown that cognitive and behavioral symptoms are common in the initial days or weeks after injury (Ponsford et al., 2000).

## Epidemiology of Traumatic Brain Injury

**FIGURE 1:**
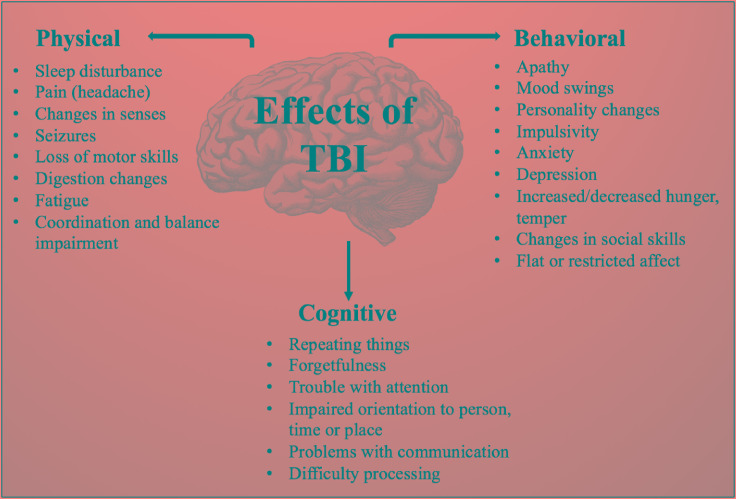
Physical, behavioral, and cognitive effects of TBI.

Brain injury occurs in two stages, eventually leading to impairment of behavioral, physical and cognitive function (Figure 1) (Hellewell et al., 2010). Primary damage results from mechanical damage associated with impaired cerebral blood flow, impaired metabolism (Stiefel et al., 2005), increased anaerobic glycolysis, accumulation of lactic acid, increase in membrane permeability, edema formation, ATP depletion and failure of energy-dependent membrane ion pumps (Werner and Engelhard, 2007). Focal injuries, a consequence of direct impact on the brain, result in tissue compression at the site of impact and are worse in cases of severe TBI (Martin, 2016). Subsequent secondary damage, related to disruption of cellular processes (Xiong et al., 2013), is characterized by membrane depolarization, excessive release of excitatory neurotransmitters, activation of NMDA and voltage-dependent Ca^2+^ and Na^+^ channels (Werner and Engelhard, 2007). The sustained influx of Ca^2+^ results in an accumulation in mitochondria causing metabolic dysfunction and energy failure (Iverson, 2005). As part of this secondary damage, several apoptotic and inflammatory pathways are activated resulting in a shift in the balance from anti-apoptotic to pro-apoptotic protein synthesis machinery (Lotocki et al., 2003; Loane et al., 2009). Overproduction of reactive oxygen species (ROS), a major cause of secondary damage from TBI, leads to mitochondrial dysfunction, damaging the cell itself (Hiebert et al., 2015). Additionally, caspase, translocase and endonuclease activation initiates progressive structural changes of biological membranes and the nucleosomal DNA (Werner and Engelhard, 2007).

Following TBI, amyloid-beta (Aβ) deposits, a hallmark of AD, have been detected histologically in young TBI patients (Schwetye et al., 2010) and in 30% of patients who die within months or years following TBI (Johnson et al., 2010). In mouse models of TBI, Aβ accumulations and increased phospho-tau immunoreactivity was observed at 24 h and up to 7 days post TBI (Tran et al., 2011). Tau levels in cerebral spinal fluid of TBI patients was found to be 1000-fold higher than that seen in controls (Liliang et al., 2010). TBI is also reported to alter the ubiquitin-proteasome system as seen by decreased levels of mRNA of ubiquitin in rats that sustained TBI (Yao et al., 2007). Hypoxia in association with hypertension occurs in about 46% of people following a TBI event (Huang et al., 2010). Hypoxia also facilitates pathogenesis of AD, upregulates accumulation of Aβ and increases hyperphosphorylation of tau promoting degeneration of neurons (Zhang and Le, 2010). Due to the large number of pathologies related to hypoxia, understanding the downstream genes that alter these hypoxia-induced processes could help develop new therapeutic strategies for management of TBI.

Patients recovering from TBI are prone to sleep-wake cycle disturbances (Baumann, 2012) which is consistent with the alteration of circadian gene expression patterns seen in rats inflicted with TBI (Boone et al., 2012). Using a weight-drop model of concussion, mice subjected to rmTBI showed impaired balance and spatial memory that persisted up to 3 months after injury (Mannix et al., 2014; Tucker et al., 2016). Such balance and coordination deficits are also described in athletes who have experienced concussive or repetitive mild brain injuries (Guskiewicz and Mihalik, 2011; Tucker et al., 2016). RNA-seq analysis of hippocampus (a main site of cognitive dysfunction in TBI pathology) and leukocyte samples from rats exposed to TBI by a fluid percussion injury (FPI) model demonstrates that TBI affects alternative splicing of genes involved in diverse functions (Meng et al., 2017). Single-cell sequencing analysis of the hippocampus from male C57BL/6 J (B6) mice inflicted with FPI identified astrocytes, oligodendrocytes and neuronal cell types as having the largest number of differentially expressed genes resulting from mTBI (Arneson et al., 2018).

Although mechanisms that lead to cell death after TBI have been analyzed extensively, little is known about how surrounding cells might mediate this cell death. The complex nature of TBI pathology, both in terms of the severity and distribution of injury to the brain and the brain’s response to injury, make the task of developing therapies a big challenge. Therefore, small animal models including rodents, flies and zebrafish can be very useful for initial characterization and identification of potential therapeutic targets which can then be interrogated in larger models for a more successful translation to human research.

## Current Models and Model Organisms Used for TBI

In view of the heterogeneous nature of brain injury pathology, several large and small animal TBI models have been developed. One of the earliest TBI models, the freeze lesion model, was used in cats, dogs and macaque monkeys (Vink, 2018). Larger animals like pigs and sheep have also been used for TBI research (Sorby-Adams et al., 2018). The physical size of the brain in these models facilitates clinically relevant monitoring of variables such as intra-cranial pressure, brain tissue oxygen content and cerebral blood flow that are also assessed in human TBI (Vink, 2018). Although these models accurately mimic human physiology (Sorby-Adams et al., 2018), there are several short-comings of using them in research. Inflicting brain injury in these models requires surgical procedures which are technically demanding and time-consuming (Sorby-Adams et al., 2018). Furthermore, longer life-span in these organisms also means considerably longer time required for study. Large animal model studies also demand access to specialized housing facilities and imaging equipment that can accommodate these animals (Dai et al., 2018).

For these reasons, rodents have become popular as models for TBI research. Rodents also share great similarity with human brains and are easier to purchase and maintain than larger animals. TBI models like FPI (Dixon et al., 1987), cortical impact injury (CCI) (Dixon et al., 1991), weight drop–impact acceleration injury (Marmarou et al., 1994) and blast injury (Leung et al., 2008) are most widely used in rodent TBI research. Recently, fruit flies and zebrafish have gained attention in TBI research because of several advantages that they offer over, like availability of an extensive genetic toolkit (Gistelinck et al., 2012), short lifespan, low cost and standardized outcome measurements (Xiong et al., 2013). These models are all targeted at improving our comprehension of the complex deleterious molecular cascades.

### Fluid Percussion Injury (FPI) Models

**FIGURE 2:**
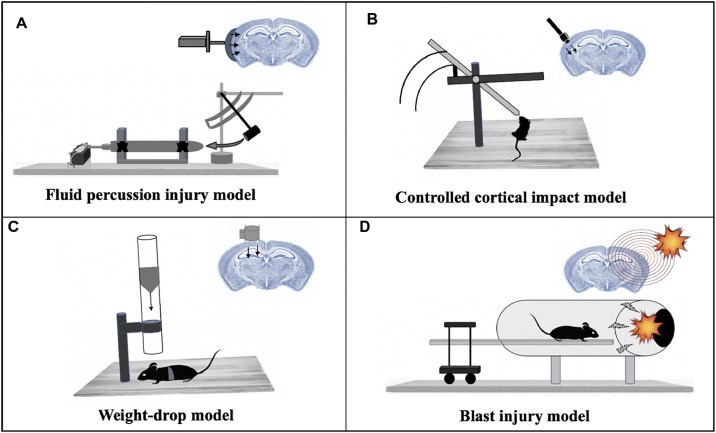
Mammalian models of TBI. **(A)** Fluid percussion injury model: *A rapid fluid pulse injection is used to cause injury directly onto the surface of the dura.* **(B)** Controlled cortical impact model: Uses an electromagnetic device to permeate the brain at a known distance and velocity. **(C)** Weight-drop model: Releases a free weight directly onto the brain. **(D)** Blast injury model: Injury caused by primary injury of blast.

The most common TBI model, FPI has been successfully applied in several animal models including rabbit, cat, rat, mouse and pig (Millen et al., 1985; Hayes et al., 1987; Marmarou and Shima, 1990; Thibault et al., 1992). The injury can be applied over the sagittal suture (midline FPI) or over the parietal cortex (lateral FPI) (Eakin et al., 2015). A fluid pulse (20 msec) is applied directly onto the surface of the dura resulting in a brief deformation of the brain tissue (Eakin et al., 2015; Figure 2A). The severity and location of the injury caused by the FPI model can be altered to reproduce neurological impairments associated with both focal and diffuse injury in humans (Dixon et al., 1987). Other aspects of human TBI that are reproduced by FPI are bradycardia, hemorrhaging at the gray – white matter interface, increased plasma glucose levels, hypertension and suppression of electroencephalogram amplitude related to the magnitude of the head injury (Cortez et al., 1989). In rodent models, FPI has been shown to produce cognitive deficits that can last for weeks to months post-injury (Hamm et al., 1993; Pierce et al., 1998). Since FPI is used widely, it is easy to compare results and outcome between laboratories but adjustments to the device are required based on the model and size of the animal.

### Controlled Cortical Impact (CCI) Injury Model

The CCI model uses an electromagnetic impactor device to produce a mechanical deformation of the cortex (Washington et al., 2012) which can be modified by changing the depth, velocity and dwell time of the impactor allowing the location of the injury to be controlled (Dixon et al., 1991; Smith et al., 1995; Manley et al., 2006; King et al., 2010; Dean et al., 2017). While leaving the dura mater intact, the impact results in a focal brain contusion injury characterized by cortical tissue loss, acute subdural hematoma, axonal injury, concussion, blood–brain barrier (BBB) dysfunction and even coma (Xiong et al., 2013; Dean et al., 2017) (Figure 2B). The CCI model has been applied to ferrets (Robie et al., 2017), rats (Dixon et al., 1991), mice (Smith et al., 1995), swine (Manley et al., 2006) and monkeys (King et al., 2010). Cognitive impairments are observed in mice and rats (Fox et al., 1998; Marklund and Hillered, 2011; Washington et al., 2012) and deficits in emotional behavior were observed in mice post-injury by CCI (Kochanek et al., 2002). The CCI swine model also generates an injury with similar pathological features as seen in human TBI (Manley et al., 2006), thus providing an opportunity to collect data post-injury in a setting similar to the intensive care unit, making the translation of animal data to human studies more relatable.

### Weight Drop–Impact Acceleration Injury

In the weight drop model, injury is caused by a free-falling, guided weight (Figure 2C) impacting an exposed skull (with or without craniotomy). Injury severity can be adjusted by altering the height and mass of the weight (Morales et al., 2005). A weight-drop model designed for rats has been shown to deliver injuries that progress from hemorrhages under the injured cortex to the development of a necrotic cavity in 24 h after injury (Feeney et al., 1981). A similar TBI model was designed for rats and mice that inflicted closed head injury delivered to one side of the unprotected skull with the head being placed on a hard surface (Shohami et al., 1988). Mice injured with this closed injury model show several clinical conditions of human TBI like neurobehavioral deficits, activation of microglia and astrocytes, neurodegeneration and morphological changes (Albert-Weissenberger et al., 2012). One disadvantage of this model involves the increased probability of skull fractures at higher magnitudes of injury severity, as well as the possibility of a rebound injury (Morales et al., 2005). The rodent weight-drop model has also been adapted for mTBI in adult zebrafish. RNA-sequencing analysis at 3- and 21-days post-injury in adult zebrafish identified differentially expressed genes enriched in peak injury response pathways, CNS injury and neurodegeneration categories (Maheras et al., 2018).

### Blast-Related Traumatic Brain Injury Model

Blast-related brain injuries are among the most commonly sustained injuries by soldiers and veterans who have served at war sites. Various test methods such as open-field blasts, blast tubes and shock tubes have been designed over the years to model explosive blast injuries suffered by humans. Of these, blast-tubes (Clemedson and Criborn, 1955; Saljo et al., 2000; Bauman et al., 2009; de Lanerolle et al., 2011) are widely used in laboratory settings (Kovacs et al., 2014), wherein a blast wave (shock wave plus blast wind) is created by the detonation of an explosive charge (Risling et al., 2011; Figure 2D). In this model, the animal is fixed with a metal net to avoid head acceleration forces (Risling et al., 2011). Expression analysis showed changes in the expression of gene families including inflammation, cell death and neurotransmitters in the hippocampus after blast injuries in rats. Genes involved in neurogenesis and synaptic transmission were found to be downregulated in this study (Risling et al., 2011).

## *Drosophila* as a Model for TBI

*Drosophila melanogaster* offers several advantages like short lifespan, cost and ease of maintenance (Tan and Azzam, 2017), and similarity to human anatomy for investigation of molecular and cellular mechanisms underlying human brain diseases (Jeibmann and Paulus, 2009). *Drosophila’s* life cycle consists of 4 distinct morphological stages (embryo, larva, pupa, and adult) each catering to different modeling functions (Pandey and Nichols, 2011). The fly genome consists of 13,500 genes (Chintapalli et al., 2007) with about 70% of genes recognized in human diseases possessing a *Drosophila* homolog (St Johnston, 2002). *Drosophila* contain less genetic redundancy compared to vertebrate models making gene characterization and loss-of-function studies less complicated (McGurk et al., 2015). Flies have an internal organ system analogs to humans including a beating heart, adipose tissue (equivalent of the liver), a tubular network (analogs to lungs), an advanced musculature, an excretion system (analogs to kidneys), a complex brain (protected by a barrier) and a nervous system with glial cells (Perrimon et al., 2016). A combination of several of these factors make *Drosophila* a very powerful model for neuroscience research.

The *Drosophila* brain is very similar to that of mammals with a similar diversity of neurons and neurotransmitters (McGurk et al., 2015), making it a great tool to study neurodegenerative diseases like Huntington’s disease, amyloidotic polyneuropathy, motor neuron disease, Parkinson’s disease (PD) and Alzheimer’s disease (AD) (Moloney et al., 2010). Fly models for these diseases are generated by mis-expression of human proteins that are neuropathological hallmark lesions in brains of patients with PD (α-synuclein), AD (tau), frontotemporal dementia (FTD) and amyotrophic lateral sclerosis (TDP-43) (Feany and Bender, 2000; Wittmann et al., 2001; Jackson et al., 2002; Li et al., 2010). Mis-expression of these proteins in flies results in neurotoxicity with molecular mechanisms that appear to be largely protein or disease specific suggesting that this approach is useful (Gistelinck et al., 2012). Cellular processes involved in neurodegeneration like oxidative stress are also exhibited in *Drosophila*. Flies also mimic complex age-dependent behaviors found in humans such as impaired memory and locomotor ability (McGurk et al., 2015).

Although one of the leading causes of mortality worldwide, relatively little is known about the factors regulating molecular responses to TBI due to the lack of an effective genetic system to model conserved tissue-specific and pathway responses (Ratliff et al., 2016). *Drosophila* genetics has been instrumental to understanding the mechanisms underlying TBI-induced disruption and several groups have successfully developed models for inflicting traumatic brain injury in *Drosophila* (Katzenberger et al., 2013; Barekat et al., 2016). TBI-inflicted flies exhibit several phenotypes observed in mammalian models including activation of neuroinflammatory responses, sleep-related behavioral defects, increased phosphorylation of the human MAPT protein in the brain, disruption of intestinal barrier and induction of autophagy, thereby proving that the underlying mechanisms are conserved in both systems (Katzenberger et al., 2015a; Ratliff et al., 2016; Anderson et al., 2018). In addition, genetic factors causing intrinsic variability in the expression of genes across the human population (inter-individual variation) significantly influence functional outcome after TBI (Diaz-Arrastia and Baxter, 2006; McAllister, 2015). Understanding the genetic architecture of quantitative traits is important for therapeutic evolution but is challenging in most species. The *Drosophila melanogaster* Genetic Reference Panel (DGRP) is a collection of 205 inbred strains that present a favorable scenario for performing genome-wide association (GWA) mapping analyses to identify candidate causal genes, polymorphisms associated with them and pathways affecting quantitative traits (Mackay and Huang, 2018).

Despite its advantages, there are some limitations for using *Drosophila* as a model for human diseases. First, genomic conservation between humans and flies is 70%, thus it is possible that expression of certain genes which may be an important target of brain injury in humans are not present in the fly (Hughes et al., 2012). Second, drug delivery is difficult in this model, therefore, one cannot accurately study and predict the effect of drugs in humans (Prussing et al., 2013). Finally, there is a lack of reliable assays to study complex behaviors and cognitive ability in *Drosophila* (Sorby-Adams et al., 2018). However, the differences in fly and human systems do not necessarily stand as an obstacle in using *Drosophila* to study brain diseases as many of these short-comings have been overcome by genetic manipulation.

Several TBI fly models have been generated and characterized for TBI associated pathways. Flies inflicted with brain trauma using the high-impact trauma (HIT) device (Katzenberger et al., 2015b) exhibited ataxia, activation of the immune response, neurodegeneration and death (Katzenberger et al., 2013). A fly model of severe and mild-repetitive TBI generated using the homogenizer shows upregulation of inflammatory and autophagy responses, increase in tau phosphorylation and sleep impairment (Barekat et al., 2016). Despite considerable morphological differences between flies and mammals, flies exhibit most of the behavior impairment seen in mammalian TBI models (Katzenberger et al., 2015a; Barekat et al., 2016). These studies demonstrate the potential of flies in providing key insights in TBI-related mechanisms and pave way for providing new opportunities of therapeutic intervention.

## *Drosophila* Models of TBI

### High-Impact Trauma (HIT) Device

**FIGURE 3:**
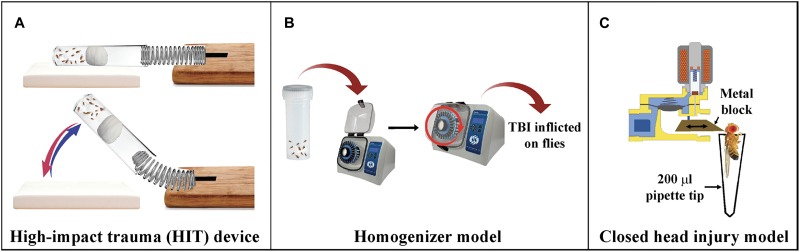
*Drosophila* models of TBI. **(A)** High-impact trauma (HIT) device: A spring attached to the wooden board on one end and a vial of flies attached to other. The vial is plugged with a cotton ball pushed deeper into the vial. The spring is deflected and released to inflict trauma to the flies when it hits the styrofoam pad. **(B)** Homogenizer model of TBI: Flies are placed in 2 ml screw cap tubes and placed in homogenizers at required speed for mild or severe TBI. **(C)** dCHI model of injury: A metal block moves forward due to the released current from the magnetic coil of the solenoid and hits the fly on top of the head.

To inflict TBI in flies, Katzenberger et al. (2013) built the HIT device (Figure 3A) (Katzenberger et al., 2013). The HIT device consists of a metal spring attached to a wooden board at one end and the free end positioned over a polyurethane/styrofoam pad. About 50–60 un-anesthetized flies are placed in a standard plastic vial connected to the spring at the free end and the flies are confined to the bottom with a cotton plug. When the spring is deflected and released, the vial contacts the pad and the acceleration-deceleration motion causes not only head trauma but full body trauma. Since force with which each fly contacts the vial is not the same for all flies in a vial, the intensity and location of the injury will vary between replicates. An immediate outcome is *ataxia*, indicating that the HIT device delivers primary injuries to the brain (Katzenberger et al., 2016). This type of variation is also common in the injuries sustained in humans from sports or vehicle crash induced TBIs.

### Bead Ruptor

To establish a model of inflicting highly reproducible levels of TBI to a large number of flies, Barekat et al. (2016) introduced the Omni Bead Ruptor-24 Homogenizer platform (Figure 3B; Barekat et al., 2016). Based on a paradigm considering intensity [meters/second, (m/s)], duration (s), and the number of injury bouts, it was determined that intensities of 5.0 m/s and higher result in nearly 100% mortality within 24 h whereas an intensity of 2.1 m/s was categorized as mild TBI. *Drosophila* injured as per this regimen exhibit several features like increased injury sensitivity, upregulation of the immune system, increased tau phosphorylation and alterations to autophagy. Additionally, brain injury in *Drosophila* is also shown to impair sleep/cycle-related behaviors, similar to what is typically observed with TBI in mammalian systems (Barekat et al., 2016).

### Closed Head Injury Model

To induce head-specific TBI to *Drosophila*, the Allada lab designed the *Drosophila* closed head injury (dCHI) device (Figure 3C; van Alphen et al., 2018). The dCHI delivers well-controlled, non-penetrating strikes to the heads of un-anesthetized flies. The device inflicts brain trauma by passage of current through a solenoid that enables forward movement of a brass block injuring the top of the fly head. The immobilized fly is aspirated in a modified 200 ml pipette tip and placed in front of the block. Injured flies exhibit many TBI phenotypes, including increased neuronal cell death, impaired sleep and motor control and increased mortality (Katzenberger et al., 2015a; Barekat et al., 2016).

## Comparison of 3 TBI Devices

Flies inflicted with trauma using the HIT device exhibit reduced lifespan, activation of innate immune response and death within 24 h (if the extent of injury exceeds a certain threshold) (Katzenberger et al., 2015b). Katzenberger et al. (2015a) also showed that death in flies inflicted with TBI using a HIT device is associated with intestinal barrier dysfunction and dysfunction of the blood-eye barrier/BBB. Overall, flies that have sustained brain trauma from the HIT device exhibit primary and secondary injuries similar to human and mammalian TBI models (Katzenberger et al., 2013, 2016; Barekat et al., 2016).

Similar to the HIT device, flies subjected to the homogenizer model also show a decrease in lifespan and an increase in sensitivity to injury with age (Katzenberger et al., 2015b). One of the key differences observed between the two models was in the mortality rate. Adult flies that have experienced trauma by the HIT device exhibit 20–25% mortality whereas mTBI-treated flies using a homogenizer exhibit minimal mortality (Katzenberger et al., 2015b; Barekat et al., 2016). Nearly 25% of w^1118^ flies injured using the HIT device had intestinal barrier dysfunction within 24 h, which directly correlates with 25% mortality (Katzenberger et al., 2015a). In contrast, <1% of mTBI treated flies exhibited the Smurf phenotype (flies exhibit blue dye throughout the body due to disruption of intestinal barrier), an effect also reflected in the minimal mortality during this time (Barekat et al., 2016). Mild brain injury has been reported to alter circadian rhythms affecting sleep maintenance which leads to excessive daytime sleepiness. The *Drosophila* mTBI model designed by Barekat et al. (2016) was able to recapitulate these findings with mTBI treated flies showing frequent awakenings during nighttime with significantly reduced duration of each nighttime sleep bout (Barekat et al., 2016). The homogenizer model for mTBI, therefore, can be considered as a good alternative to the HIT device for inflicting trauma in *Drosophila* especially with regards to reproducibility of trauma intensity and reduced variation. However, neither of these models escape full body trauma to the fly.

The Allada lab used glial targeted translating ribosome affinity purification in combination with RNA sequencing (TRAP-seq) and identified glial immune pathways as mediators of TBI effects in flies injured using dCHI (van Alphen et al., 2018). The *Drosophila* innate immune system consists of the Immunodeficiency (Imd), Toll and JAK-STAT pathways, which combat fungal and bacterial infections (Lemaitre and Hoffmann, 2007; Sudmeier et al., 2015). An upregulation of several immune genes like *alrm (astrocytic leucine-rich repeat molecule, astrocyte-specific), moody (blood brain barrier), wunen-2 (wun2, astrocyte-specific), reversed polarity (repo, pan-glial)*, and *gli (gliotactin, expressed in peripheral glia)* was seen 24 h after TBI in this study. In addition, they have also observed increased mortality and impaired negative geotaxis response, increased apoptotic cell death, impaired motor control, reduced lifespan, reduced and fragmented sleep and upregulation of stress response genes after TBI. Overall, dCHI is a good model for closed head injury in *Drosophila* especially since the trauma is restricted to the head only and is also highly reproducible. However, with dCHI, each replicate will have to be treated separately since the device can only handle one fly at a time, but the trauma is consistent between replicates.

## Summary of Transcriptomic Changes Induced by TBI

Mice and rat are the two most commonly used mammalian models for studying TBI. However, due to the short life-span, sophisticated genetics and ease of maintenance (Katzenberger et al., 2013), *Drosophila* has garnered attention as an attractive model for the study of neurodegenerative diseases. Lately, genome-wide sequencing to identify changes in gene expression induced by brain injury has also become a focus of study among researchers. RNA-seq datasets for studies on rat, mice and flies have identified several genes and gene ontology terms that are enriched in response to TBI.

In the *Rattus norvegicus* (*Rn*) study (GSE64986) (Meng et al., 2017), adult male Sprague–Dawley rats were injured using the FPI, and RNA-seq was performed on the hippocampus and leukocytes collected 7 days after injury (Meng et al., 2017). Original analyses revealed that TBI affected alternative splicing of genes involved in functions related to neurons, complement and coagulation, transcription factors, blood pressure, inflammation, mitochondria, leptin signaling, insulin signaling and extracellular matrix genes. This study also identified a large-scale switch of the DNA methylation patterns in these genes in both the hippocampus and leukocytes.

In the *Mus musculus* (*Mm*) study (GSE79441) (Zhong et al., 2016), male C57BL/6 mice, aged 12 weeks were inflicted with brain trauma using the controlled cortical impact (CCI) model. Differential expression analysis of the RNA-seq data detected 64,530 transcripts including 27,457 identified as mRNA and 37,073 as long non-coding RNA. KEGG pathway analysis showed that a total of 234 pathways were involved in this dataset with MAPK signaling pathway, NF-kappa B signaling pathway, ECM-receptor interaction, cytokine-cytokine receptor interaction, chemokine signaling pathway, phagosome, PI3K-Akt signaling pathway, cell adhesion, osteoclast differentiation, complement and coagulation cascades being the most enriched pathways.

In the *D. melanogaster* (*Dm*) study (GSE85821) (Katzenberger et al., 2016), *w^1118^* male flies aged 0–7 days and 20–27 days received 4 strikes from the HIT device (Katzenberger et al., 2015b) with 5-min inter-injury intervals. The flies were either fed on water for 2 h or food for 4 h. RNA was extracted from whole flies for sequencing after injury and feeding. Sequencing data has shown that immune response genes dominate the early transcriptional responses to injuries. In addition, age and diet affect expression of immune response genes after primary injuries. The Ruden lab subjected *w^1118^* flies to mild closed head trauma using a modified HIT device and found reduced mitochondrial activity in fly brains after 24 h (Sen et al., 2017). RNA-seq analysis on these fly heads showed selective retention (RI) of long introns. Some of the genes that exhibit RI are involved in mitochondrial metabolism and showed a significant reduction in transcript abundance (Sen et al., 2017).

Reanalysis of select RNA-seq datasets from each species (Katzenberger et al., 2016; Zhong et al., 2016; Meng et al., 2017) revealed striking similarities in the outcome post-injury. Studies in all 3 models of TBI show an upregulation of genes involved with immune response. Mice and rat data show upregulated genes associated with signaling pathways, locomotion, inflammatory response and programmed cell death. All 3 datasets show enrichment of genes associated with protein phosphorylation which is in line with studies indicating hyper-phosphorylation of microtubule-binding protein tau post injury (Stoothoff and Johnson, 2005; Olczak et al., 2017; Rubenstein et al., 2017). Genes associated with localization and cytoskeletal organization were upregulated in mouse and fly. This finding is consistent with studies indicating disruption of microtubule network, the building block of cytoskeleton and axonal degeneration post-TBI (Johnson et al., 2013).

In *Drosophila*, there is a significant upregulation of *Sid*, a nuclease gene induced in response to oxidative stress (Seong et al., 2014) and an important mediator of TBI pathology. In addition, there is also an upregulation of the gene *Grim*, involved in apoptosis of cells in the central nervous system. The fly dataset also shows upregulation of genes involved in cognition and learning-memory. Overall, there are 1626 genes upregulated in mouse, 323 genes upregulated in fly and 173 genes upregulated in rat. Of these, 32 genes are shared between mouse and fly, 48 are shared between mouse and rat, 6 are shared between fly and rat, and 2 are common in all 3 datasets. There was no significant overlap observed between down-regulated genes from all 3 datasets. However, there were several gene ontology (GO) terms that significantly overlap between the species (either fly and mouse/mouse and rat/all 3) (Table 1).

**Table 1:** Summary of transcriptomic changes induced by TBI.

GOBPID	Term	Species
GO:0002376	Immune system process	*Mm*
GO:0006955	Immune response	*Mm*
GO:0006952	Defense response	*Dm, Mm*
GO:0006954	Inflammatory response	*Mm, Rn*
GO:0006950	Response to stress	*Dm, Mm*
GO:0002682	Regulation of immune system process	*Mm*
GO:0009605	Response to external stimulus	*Mm, Rn*
GO:0002684	Positive regulation of immune system process	*Mm*
GO:0045087	Innate immune response	*Mm*
GO:0001816	Cytokine production	*Mm, Rn*
GO:0007601	Visual perception	*Rn*
GO:0050953	Sensory perception of light stimulus	*Rn*
GO:0051179	Localization	*Rn*
GO:0065008	Regulation of biological quality	*Mm, Rn*
GO:0044699	Single-organism process	*Rn*
GO:0044765	Single-organism transport	*Mm, Rn*
GO:1902578	Single-organism localization	*Mm, Rn*
GO:0006811	Ion transport	*Mm, Rn*
GO:0032879	Regulation of localization	*Mm, Rn*
GO:0048646	Anatomical structure formation involved in morphogenesis	*Mm, Rn*
GO:0010824	Regulation of centrosome duplication	*Dm*
GO:0007610	Behavior	*Dm*
GO:0044763	Single-organism cellular process	*Dm, Mm, Rn*
GO:0046394	Carboxylic acid biosynthetic process	*Dm, Mm*
GO:0009628	Response to abiotic stimulus	*Dm, Mm, Rn*
GO:0009719	Response to endogenous stimulus	*Dm, Mm, Rn*
GO:0014048	Regulation of glutamate secretion	*Dm*
GO:0046605	Regulation of centrosome cycle	*Dm*
GO:0044699	Single-organism process	*Dm, Mm, Rn*
GO:0016053	Organic acid biosynthetic process	*Dm*


*Table showing top 10 upregulated gene-ontology (GO) terms for mouse *(Mm)*, rat *(Rn)*, and fly *(Dm)* RNA-seq datasets at *p*-value < 0.05. Species column also indicates the terms that are common between any 2 or all 3 datasets. GOBPID, the ID of biological process in GO database.*

A large portion of gene expression changes commonly seen in human TBI studies are also observed in these animal models. Proteomic analysis of post-mortem human TBI brains show alterations in immune response, synaptic and mitochondrial function (Harish et al., 2015). Gene expression analysis on pericontusional tissue from TBI patients also showed differential gene expression across categories related to transcriptional control, signal transduction, immune functions, cytoskeletal development and cell cycle (Michael et al., 2005). Visual perception, inflammatory response, defense response, MAPK signaling, PI3K-Akt signaling pathway, chemokine activity and cell adhesion are some of the categories enriched in animal models and also seen to be enriched in microarray analysis of brain contusion tissue taken from 3 TBI patients (Yang et al., 2019).

These findings demonstrate that several responses related to TBI are conserved among model systems but there are also species-specific processes that are affected post-injury.

## Expression Analysis

SRA files for all 3 datasets were downloaded from Gene Expression Omnibus and converted to fastq format. Quality of paired-end RNA-seq reads was verified prior to alignment to the respective genome (Build mm9, rn6 and dm3) and tabulated across gene regions (Andrews, 2010; Dobin et al., 2013; Anders et al., 2015). Differential gene expression analysis was used to compare transcriptome changes between conditions (Robinson et al., 2010). Finally, GO enrichment analysis was performed on significant genes (logFC > |2|; *p*-value < 0.05) using DAVID [Database for annotation, visualization, and integrated discovery] (Huang da et al., 2009a,b). Fly and rat gene names were converted to homologous mouse genes in order to compare the overlapping genes between the three species.

## Future Directions

A variety of animal models have been developed that mimic the different injury mechanisms associated with human TBI. In spite of all these advancements, therapeutic strategies for treatment of TBI are limited since current diagnosis relies on identifying symptoms and monitoring trauma patients. The Brain trauma foundation recommends decompressive craniotomy, hypothermia, hyperosmolar therapy, cerebrospinal fluid drainage and ventilation therapies for management of severe TBI (Carney et al., 2017). Achieving a therapeutic breakthrough in TBI still requires the development of new clinically relevant models, refinements of established models and functional tests, consideration of systemic insults and searching for specific and sensitive biomarkers. In addition, more research into the effect of age, sex and genotype on the outcome of TBI is necessary.

*Drosophila* has proven to be a useful model to study not only neurodegenerative diseases but also disorders associated with other systems. Single-cell sequencing done on adult *Drosophila* brain has been able to present a brain atlas that covers all cells in normal brain and how it changes over the lifespan of a fly (Davie et al., 2018). Similar approaches can be adapted to identify cell sub-populations that are affected by TBI to help design therapeutic targets for improving patient outcome after injury.

So far, none of the studies have taken full advantage of the sophisticated genetics and short lifespan the *Drosophila* model. Such studies could include, for instance, genetic screens for mutations that make flies sensitive or resistant to TBI, thus provide novel targets for therapeutics. Additionally, drug screens can be done in the fly model to identify drugs that protect against the deleterious effects of TBI in flies, and possibly in humans.

## Data Availability

Publicly available datasets (GSE64986, GSE79441, and GSE85821) were analyzed in this study and can be found at www.ncbi.nlm.nih.gov/geo.

## Author Contributions

DR is the principal investigator and conceived the project. ES and KG conceptualized the content. ES performed the data analysis and wrote the manuscript. KG edited the manuscript and assisted with data analysis.

## Conflict of Interest Statement

The authors declare that the research was conducted in the absence of any commercial or financial relationships that could be construed as a potential conflict of interest.
